# Role of the inositol 1,4,5-trisphosphate receptor/Ca^2+^-release channel in autophagy

**DOI:** 10.1186/1478-811X-10-17

**Published:** 2012-07-06

**Authors:** Jan B Parys, Jean-Paul Decuypere, Geert Bultynck

**Affiliations:** 1 Laboratory of Molecular and Cellular Signaling, Department of Cellular and Molecular Medicine, KU Leuven, Campus Gasthuisberg O/N-1 bus 802, Herestraat 49, BE-3000, Leuven, Belgium

**Keywords:** Ca^2+^, Autophagy, Inositol 1,4,5-trisphosphate receptor, Beclin 1, Bcl-2, AMP-activated kinase, Mammalian target of rapamycin, Calmodulin-dependent kinase kinase β

## Abstract

Autophagy is an important cell-biological process responsible for the disposal of long-lived proteins, protein aggregates, defective organelles and intracellular pathogens. It is activated in response to cellular stress and plays a role in development, cell differentiation, and ageing. Moreover, it has been shown to be involved in different pathologies, including cancer and neurodegenerative diseases. It is a long standing issue whether and how the Ca^2+^ ion is involved in its regulation. The role of the inositol 1,4,5-trisphosphate receptor, the main intracellular Ca^2+^-release channel, in apoptosis is well recognized, but its role in autophagy only recently emerged and is therefore much less well understood. Positive as well as negative effects on autophagy have been reported for both the inositol 1,4,5-trisphosphate receptor and Ca^2+^. This review will critically present the evidence for a role of the inositol 1,4,5-trisphosphate receptor/Ca^2+^-release channel in autophagy and will demonstrate that depending on the cellular conditions it can either suppress or promote autophagy. Suppression occurs through Ca^2+^ signals directed to the mitochondria, fueling ATP production and decreasing AMP-activated kinase activity. In contrast, Ca^2+^-induced autophagy can be mediated by several pathways including calmodulin-dependent kinase kinase β, calmodulin-dependent kinase I, protein kinase C θ, and/or extracellular signal-regulated kinase.

## Review

### What is autophagy?

Autophagy is the name for a group of lysosomal degradation processes conserved throughout evolution [1,2]. It is responsible for the disposal of cellular material that cannot be degraded by the ubiquitin-proteasome system, like long-lived proteins, other macromolecules and even entire organelles. Depending on the delivery mechanism of this material to the lysosomes, autophagy is divided into three main types: microautophagy, chaperone-mediated autophagy and macroautophagy [2,3].

The latter is the best-studied form of autophagy and involves the formation of a typical double-membrane cistern, named phagophore, which surrounds and ultimately engulfs the cytoplasmic material to be degraded. The resulting vesicle is the autophagosome, which can fuse with late endosomes or lysosomes, leading to the breakdown of its content [1,4]. Because macroautophagy is the main focus of this review, it will further be referred to as autophagy.

Appropriate autophagy levels are not only needed for cellular homeostasis (removing of aggregated proteins and damaged organelles) [5,6] but also for development, cell differentiation, ageing and tumor suppression [1,6,7].

Depending on the type of stress faced by cells, autophagy can either provide the cell with building blocks and energy (e.g. during starvation) or help the cell to cope with potentially damaging elements (e.g. aggregated proteins, viral infection and other intracellular pathogens) [1,4,6,8,9]. Thus, autophagy ensures cell survival, but if the stress persists for a longer time, it can lead to cell death, also termed programmed cell death type 2 [10]. Cell death is however not the normal outcome of autophagy, and the concept has been proposed that cell death may occur along *with* autophagy rather than executed *by* autophagy [11,12].

Impaired or altered autophagic flux has been implicated in several pathologies, including cancer and neurodegenerative disorders [1,6,8].

**Figure 1:**
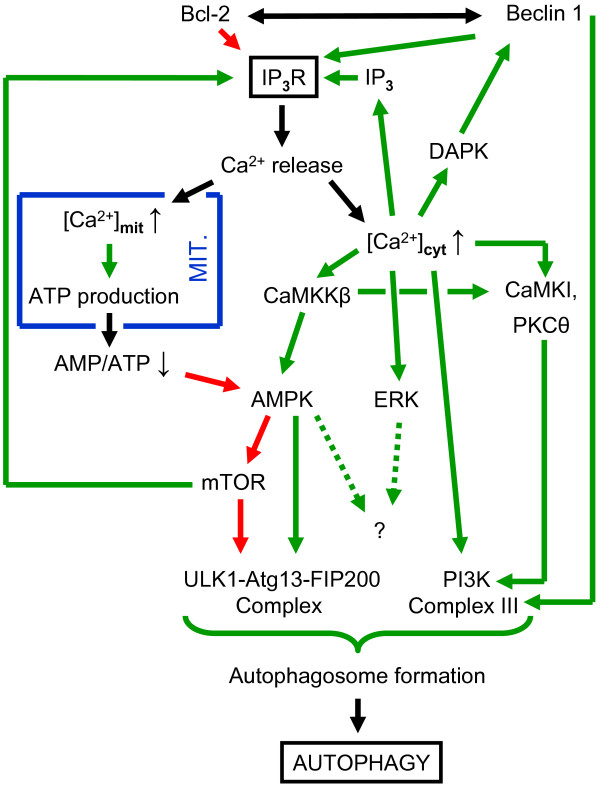
**Relation between IP**_**3**_**R and autophagy.** IP_3_-induced Ca^2+^ release towards the mitochondria promotes ATP production, inhibition of AMPK and thus stimulation of mTOR activity. mTOR can stimulate the IP_3_Rs by direct phosphorylation. Inhibition of mTOR leads to the formation of the ULK1-Atg13-FIP200 complex and autophagy. In addition, the IP_3_R might act as scaffold for Bcl-2 and Beclin 1, thereby promoting the inhibition of Beclin 1 by Bcl-2. Beclin 1 promotes the formation of the PtdIns3K Complex III. Both complexes are necessary for the formation of the phagophore and subsequent autophagy. Ca^2+^ (and calmodulin) can activate multiple systems in the cell including CaMKKβ, ERK, CaMKI, PKCθ, DAPK, which phosphorylates Beclin 1, thereby mediating its dissociation from Bcl-2, and putatively also Vps34, a component of the PtdIns3K Complex III. The blue box indicates the mitochondrial (Mit.) compartment. Arrows indicate the flow of the signals; a green arrow indicates stimulation; a red arrow indicates inhibition; a double arrow indicates an interaction; a broken arrow indicates a mechanism that remains to be further defined. For more detailed explanation, see text.

In order to avoid uncontrolled or excessive levels of autophagy, the process is tightly regulated. More than 30 autophagy genes (atg) have hereby been identified as essential regulators [1,13]. The mammalian target of rapamycin (mTOR) is an important upstream negative regulator of the canonical autophagy pathway. In normal conditions, mTOR hyperphosphorylates Atg13, thereby inhibiting its activity. In conditions where mTOR is inhibited, e.g. subsequently to activation of the upstream AMP-activated kinase (AMPK), the resulting active Atg13 forms a complex with Atg1/Unc-51-like kinase 1 (ULK1) and FIP200, the focal adhesion kinase family interacting protein of 200 kDa [14]. In addition, mTOR also directly phosphorylates ULK1 at S757, thereby preventing the interaction between AMPK and ULK1. During starvation, AMPK directly activates ULK1 by phosphorylation on S317 and S777 [15,16]. The ULK1 complex together with the class III phosphatidylinositol 3-kinase complex (PtdIns3K Complex III), which mainly consists of PtdIns3K (Vps34), Vps15, Atg6/Beclin 1 and Atg14/Barkor [17], are necessary for phagophore formation (Figure 1).

Beclin 1 plays a central and critical role in these initial steps as a platform protein, recruiting other regulatory proteins to the PtdIns3K Complex III [18]. It was originally identified as a 60 kDa Bcl-2-interacting protein [19]. Structurally, it consists of an N-terminal domain containing a BH3 domain, a central coiled-coil domain, and a C-terminal evolutionarily conserved domain. Although other functions are possible [20], its best-characterized function is its role in autophagy; moreover, in contrast to the other BH3-only proteins, it does not promote apoptosis [13,21,22]. In normal conditions, Beclin 1 is neutralized by binding through its BH3 domain to the hydrophobic cleft of the anti-apoptotic Bcl-2-protein family members Bcl-2, Bcl-Xl, Mcl-1 and Bcl-w. Their interaction can be dynamically regulated by various mechanisms, allowing the release of Beclin 1 and subsequent activation of the PtdIns3K Complex III during autophagy-inducing conditions [23]. A first mechanism involves phosphorylation of either Bcl-2 or Beclin 1. Bcl-2 can be phosphorylated by c-Jun NH_2_-terminal kinase-1 (JNK1) [24], and Beclin 1 by death-associated protein kinase (DAPK) [25]. Either phosphorylation opposes the interaction between the two proteins. Also a number of regulatory proteins can modulate the interaction of Beclin 1 with Bcl-2 [26]. For instance, under hypoxic conditions, BNIP3 will bind Bcl-2 and Bcl-Xl through its BH3 domain, thereby dissociating Beclin 1 from them and triggering autophagy [27]. After starvation and reactive oxygen species production, HMGB1, the High Mobility Group Box 1 protein, translocates to the cytosol, where it can disrupt the Beclin 1/Bcl-2 complex and thus induce autophagy [28]. Another protein that can play a role in this process is Nutrient-deprivation Autophagy Factor-1 (NAF-1). NAF-1 binds both Bcl-2 and the inositol 1,4,5-trisphosphate (IP_3_) receptor (IP_3_R), stabilizing the Beclin 1/Bcl-2 interaction and inhibiting the induction of autophagy [29]. In addition, other Beclin 1-associated proteins also can enhance (Ambra-1, UVRAG or Bif-1) or inhibit (Rubicon) Beclin 1’s autophagy-stimulating functions [17]. Furthermore, recent work identified the importance of the intracellular localization and membrane recruitment of Beclin 1 for its role in autophagy. It appears that although both Beclin 1 and Bcl-2 are also found at the mitochondria, inhibition of Beclin 1’s function in autophagy mainly depends on Bcl-2 that is located at the endoplasmic reticulum (ER) [26]. In addition, membrane anchoring of Beclin 1 seems a critical factor in its ability to induce autophagy. Beclin 1 was recently shown to bind to lipid membranes containing either cardiolipin or other lipids favoring a negative curvature, which may be related to the formation of omegasomes, the early precursors of autophagosomes [30]. Three aromatic amino acids (F359, F360 and W361) responsible for this interaction have been identified and seemed critical for Beclin 1’s role in autophagy. Indeed, Beclin 1 mutants lacking these three aromatic residues fail to bind lipid membranes and are impaired in their ability to rescue autophagy in Beclin 1-deficient cells.

Once the phagophore is formed, its further elongation depends on the formation of the Atg5-Atg12-Atg16L1 complex [31] and the lipidation with phosphatidylethanolamine of microtubule-associated protein 1 light chain 3 (Atg8/LC3) to LC3-II. The lipid tail enables LC3-II insertion into the membrane [31]. The resulting autophagosomes are subsequently transported along microtubules via a dynein-dependent mechanism. Final fusion with endosomes and lysosomes is regulated by ESCRTIII, SNAREs, Rab7 and class C Vps proteins [32].

Interestingly, the processes of apoptotic cell death and of pro-survival autophagy are interrelated in a complex way. They can have antagonistic, additive or even synergistic effects, depending on cell type and conditions [6]. This interplay is also evident from the molecular interactions occurring between apoptosis- and autophagy-related proteins, including the Beclin 1/Bcl-2 interaction. Also the pro-apoptotic tumor suppressor p53 has regulatory effects on autophagy, while p62 is not only involved in the delivery of cargo to the autophagosomes, but also in caspase 8 activation [6]. Many Atg proteins also serve as caspase substrates [33], whereby the cleaved C-terminal fragment of Beclin 1 even gains pro-apoptotic functions [34]. A similar switch from pro-autophagic to pro-apoptotic functions were found upon calpain-mediated cleavage of Atg5 [35], indicating many levels of interaction between those two pathways. In addition, Ca^2+^ is a well-known regulator of many intracellular processes, including apoptosis [36,37]. Especially the IP_3_R, a Ca^2+^ channel mainly located in the ER, plays hereby a crucial role [38,39]. More recently, it appeared that the IP_3_R may also play an important role in the control of autophagy, though the available data on the role of Ca^2+^ and the IP_3_R in autophagy are at least partially contradictory [40,41].

In this review, we will therefore critically review the available evidence on the role of the ER Ca^2+^ store and in particular of the IP_3_R in the regulation of autophagy. While the role of the IP_3_R in controlling apoptosis is already well-established, its function in regulating autophagy only quite recently emerged. The available data however not only support a role for the IP_3_R and for IP_3_-induced Ca^2+^ release in the control of autophagy, but indicate that this role can depend on the exact conditions and so either has a preventing or an enhancing role with respect to autophagy.

### Role of the IP_3_R in autophagy

#### Importance of intracellular Ca^2+^ in autophagy

The role of Ca^2+^ in the regulation of autophagy has been investigated since 1993 [42], and the first study already indicated a complex role for Ca^2+^ as both an increase and a decrease in the cytosolic [Ca^2+^ suppressed autophagy. This complexity was not directly resolved as further reports suggested that intracellular Ca^2+^ signaling and Ca^2+^-handling proteins inhibited autophagy, while other reports indicated a stimulatory role for Ca^2+^ (reviewed in [40]). Although Ca^2+^ release from the ER has hereby a major role, it is important to note that also other intracellular compartments may contribute to the control of autophagic flux by Ca^2+^, including the lysosomes. For instance, in rat astrocytes, nicotinic acid adenine dinucleotide phosphate (NAADP) has been shown to trigger Ca^2+^ release from these acidic compartments via the two-pore channels and so to induce autophagy [43]. Interestingly, the leucine-rich repeat kinase-2 can induce autophagy by, directly or indirectly, activating this pathway, leading to an increase in cytosolic [Ca^2+^, possibly also activating Ca^2+^-induced Ca^2+^ release from the ER, and finally activating calmodulin-dependent kinase kinase β (CaMKKβ) and AMPK [44].

#### The IP_3_R

In any case, there is no doubt that intracellular Ca^2+^ signals affect autophagy with hereby a prominent role for the ER as the main intracellular Ca^2+^ store and the IP_3_R as the most ubiquitously expressed intracellular Ca^2+^-release channel. Three genes encode an IP_3_R, leading to the expression of three IP_3_R isoforms (IP_3_R1, IP_3_R2, IP_3_R3). All IP_3_R isoforms are activated upon cell stimulation, phospholipase C activation and subsequent IP_3_ production. IP_3_ diffuses into the cytoplasm and binds and activates the IP_3_R, leading to IP_3_-induced Ca^2+^ release. The IP_3_R isoforms vary in a number of properties, including their affinity for IP_3_ and their regulation mechanisms. Main regulatory factors are the cytosolic and the luminal [Ca^2+^, ATP, their phosphorylation status and their interaction with regulatory proteins [45-47]. The subsequent complex spatio-temporal Ca^2+^ signals occurring in the cell regulate many intracellular processes, including cell death [36-39,48].

#### The IP_3_R suppresses autophagy

A first study implicating the role of the IP_3_R in autophagy was based on the use of Li^+^[49]. Li^+^ induced autophagy by inhibiting inositol monophosphatase, and subsequently decreasing IP_3_ levels. Autophagy was induced in an mTOR-independent manner, as no decrease in phosphorylation of mTOR substrates was observed, and it was proposed that the IP_3_R acted as an inhibitor of autophagy. This finding was confirmed in another study, demonstrating that in HeLa cells chemical inhibition of IP_3_Rs with xestospongin B (XeB), a potent and selective IP_3_R antagonist [50] or suppression of IP_3_R expression using siRNA also induced autophagy [51].

To further investigate the role of the IP_3_R, several groups investigated the properties of the IP_3_R triple knock out chicken DT40 B lymphocytes (TKO cells) originally developed by T. Kurosaki [52]. These cells displayed higher levels of autophagic markers than their wild-type counterparts in two studies [53,54], but not in a third one [55]. The variation between those results may however be due to the exact experimental conditions, as these cells appear extremely sensitive to nutrient supply [40]. Anyway, in both studies demonstrating higher basal autophagy levels in the TKO cells, heterologous expression of either IP_3_R1 or IP_3_R3, but not of the type 2 ryanodine receptor, another ER Ca^2+^-release channel, suppressed autophagic levels [53,54].

It was proposed that the control of autophagy by the IP_3_R depended on the binding of Beclin 1 to the IP_3_R [55]. In this scaffolding model, the IP_3_R facilitates the binding of Beclin 1 to Bcl-2 by recruiting both proteins. This model is attractive, because Beclin 1 and Bcl-2 have been proposed to target distinct IP_3_R regions with Beclin 1 binding to the IP_3_-binding domain [55] and Bcl-2 predominantly binding in the middle of the modulatory and transducing domain [56,57]. Moreover, it was proposed that XeB would dissociate Beclin 1 from the IP_3_R/Bcl-2 complex and so induce autophagy [55]. According to this model, the IP_3_R Ca^2+^-channel function would not be involved. Correlating with this, siRNA-mediated knock down of Beclin 1 did neither affect histamine-induced Ca^2+^ release nor the steady-state ER Ca^2+^ content [55].

Control of autophagy by the IP_3_R but independently of IP_3_-induced Ca^2+^ release was however not confirmed in other studies. TKO cells expressing channel-dead IP_3_R mutants (IP_3_R1 D2550A, IP_3_R3 D2550A or T2591A) had, in contrast to those expressing wild-type IP_3_R1 or IP_3_R3, similar levels of autophagic markers as control TKO cells [53,54]. This strongly suggests that Ca^2+^ release through the IP_3_R is critical for the inhibition of autophagy by the IP_3_R, and it was proposed that this was related to a regulation of mTOR activity [53].

The group of K. Foskett performed a very detailed study to clarify the inhibitory effect of the IP_3_R on autophagy induction [54]. It is well established that a fraction of the IP_3_Rs are present in ER domains forming close associations with the mitochondria [58]. These domains are structurally stabilized by various proteins, and allow efficient transfer of Ca^2+^ ions from the ER to the mitochondria [48,59-61]. The study by Cárdenas et al. [54] showed in TKO cells increased glucose and decreased O_2_ consumption, pyruvate dehydrogenase inhibition and AMPK activation. These observations suggest a mechanism in which constitutive Ca^2+^ release through IP_3_Rs fuels into the mitochondria, thereby augmenting mitochondrial bio-energetics and ATP production [54]. Also in neuroblastoma cells these essential Ca^2+^ signals could be abolished via siRNA-mediated knock down or XeB-mediated inhibition of IP_3_Rs, leading to a decreased ATP production, an increased AMP/ATP ratio and subsequent AMPK activation and autophagy stimulation. As mTOR activation appeared unaffected, a non-canonical AMPK-dependent stimulation of autophagy was proposed [54]. A possible pathway is e.g. via direct phosphorylation of ULK1 by AMPK [15].

In follow-up on these results, recent data indicate that Bax inhibitor-1 (BI-1) overexpression promotes autophagy by decreasing ER Ca^2+^ store content, decreasing thereby IP_3_R-mediated Ca^2+^ transfer to the mitochondria, O_2_ consumption, and ATP production, and thus leading to AMPK stimulation and autophagy induction [62]. Moreover, BI-1 overexpression in TKO cells was without effect, further demonstrating that BI-1 induced autophagy via a pathway requiring the IP_3_R.

Although it is not yet clearly established by which pathway AMPK activation leads to autophagy induction in response to IP_3_R inhibition, it is clear that conditions suppressing IP_3_R activity cause autophagy induction via a mitochondrial pathway [41]. Under a normal situation however, basal autophagy levels are kept minimal through a Ca^2+^- and IP_3_R-dependent mechanism, although an additional scaffold function for the IP_3_R cannot be excluded. Moreover, the relation between the IP_3_R and mTOR activity may form a feedback loop, as it was shown that the various IP_3_R isoforms can be phosphorylated and stimulated by mTOR [63-65] (Figure 1).

#### The IP_3_R can promote autophagy

In contrast to the previous, other results indicated that intracellular Ca^2+^ signaling, and hence potentially the IP_3_R, can activate autophagy. Several of those studies [66-69] were based on the use of thapsigargin, a potent sarco/endoplasmic reticulum Ca^2+^- ATPase inhibitor. Treatment of cells with thapsigargin results in an increase in their cytosolic [Ca^2+^ and increased autophagy. Similar results were found for other treatments that increased [Ca^2+^ (reviewed in [40]). As the induction of autophagy by Ca^2+^-mobilizing agentia was counteracted by BAPTA-AM but was not affected in cells deficient for proteins involved in the unfolded protein response (IRE1α, eIF2α, ATF6) [67], it can be assumed that the induction of the autophagic flux was not consecutively to ER stress, an event triggered by the depletion of the ER Ca^2+^ stores [40]. Although this does not exclude a priori a role for Ca^2+^ originating from other compartments, the existing data support an important role for the ER Ca^2+^ stores in autophagy induction and in a number of studies a direct role for the IP_3_R herein was even presented.

In a first study, it was assumed that the Cd^2+^-induced autophagy was linked to Ca^2+^ mobilization via IP_3_Rs [70]. The involvement of IP_3_Rs was deduced from indirect evidence based on the use of the IP_3_R inhibitor 2-aminoethoxydiphenyl borate (2-APB) and the siRNA-mediated knock down of Ca^2+^/calmodulin-dependent phosphatase calcineurin putatively acting on the IP_3_R. However, 2-ABP is not specific for the IP_3_R [71,72], and it has not yet been clarified in which circumstances calcineurin exactly regulates IP_3_R activity [46,73].

A second study indicating a role for the IP_3_R in autophagy was performed in the slime mold Dictyostelium discoideum [68]. Because this organism lacks the apoptotic machinery, autophagy can be studied in the absence of any interference by apoptosis [74]. Importantly, in this organism, the differentiation factor DIF-1 led from starvation-induced autophagy to autophagic cell death, which did not occur when the unique IP_3_R gene was inactivated by random insertional mutagenesis or when IP_3_-induced Ca^2+^ signaling was blocked by BAPTA-AM [68].

Finally, in mammalian cell lines, we recently determined that the induction of autophagy triggered by nutrient starvation occurred concomitantly with a remodeling of the proteins involved in ER Ca^2+^ homeostasis and dynamics, thereby sensitizing multiple Ca^2+^-handling mechanisms. During the initial phase of the autophagy response, the levels of the Ca^2+^-binding chaperones calreticulin and GRP78/BiP increased and ER Ca^2+^ leak decreased, resulting in an elevation of the steady state ER Ca^2+^ levels, while the sensitivity of the IP_3_R towards IP_3_ increased [75]. This sensitization of the Ca^2+^-flux properties of the IP_3_R was not only indirectly due to the increased ER Ca^2+^-store content, but also involved direct effects through increased Beclin 1 binding to the IP_3_R. Functional experiments suggested that predominantly the N-terminal part of Beclin 1, containing the BH3 domain, was involved in this stimulation. Moreover, Beclin 1 appears to interact with the N-terminal part of the IP_3_R, especially in its suppressor domain (a.a. 1–225) [75]. This domain regulates at the one hand the affinity of the IP_3_R for IP_3_[76] and at the other hand interacts with and regulates its C-terminal Ca^2+^-channel domain [77]. Importantly this sensitization of the IP_3_R is essential for the proper induction of autophagy upon nutrient starvation, as cells loaded with BAPTA-AM displayed an impaired autophagic flux [75]. This is in agreement with results from other groups demonstrating that autophagy induction in response to various autophagic triggers, including nutrient deprivation, was also inhibited by BAPTA-AM [78-81]. Results obtained with the IP_3_R inhibitor XeB were more complex, because XeB induced autophagic flux in normal cells, but suppressed autophagic flux in starved cells [75]. This points towards a dual role for IP_3_R function in autophagy depending on the cellular condition. In normal cells, IP_3_Rs suppress autophagic flux by fueling Ca^2+^ into the mitochondria to sustain ATP production, thereby preventing AMPK activity [41,54]. In nutrient-deprived cells, however, IP_3_Rs are required to promote Ca^2+^-signaling events that are critical for up-regulating autophagic flux [40,75].

As Bcl-2 can suppress IP_3_-induced Ca^2+^ release [56,82,83], it may be argued that IP_3_R sensitization by Beclin 1 is an indirect effect, due to its effects on Bcl-2, e.g. by dissociating Bcl-2 from IP_3_Rs. However, a Beclin 1 mutant unable to bind Bcl-2 [84] remained able to sensitize IP_3_-induced Ca^2+^ release in vitro, indicating that these events were not due to a suppression of the inhibitory effect of Bcl-2. Nevertheless, in a cellular context, Bcl-2 seems to play an important role in tethering Beclin 1 at the ER membranes in the proximity of the IP_3_R channel [75].

#### Autophagy can be positively or negatively regulated by the IP_3_R

Taken together these various results indicate a complex action of the IP_3_R in autophagy regulation, whereby depending on the state of the cells IP_3_-induced Ca^2+^ release can suppress or promote autophagy (Figure 1). This complex behavior probably also explains in part the contradictory results obtained in cells treated with thapsigargin or BAPTA-AM. Indeed, differing cellular conditions, concentrations of the applied chemicals and incubation times could underlie the different results obtained in different studies [40]. Finally, also the localization of the IP_3_Rs and the subcellular localization of the resulting Ca^2+^ signals (cytosol or mitochondria) may determine the specific outcome on autophagy. In addition, it can be expected that regulators of the IP_3_R may impinge on the cellular autophagy levels by modulating IP_3_-induced Ca^2+^ release. Main regulators in this context are obviously Beclin 1 and Bcl-2, though their exact regulation by associated proteins or phosphorylation events remains to be explored. Moreover, as these same proteins control apoptosis [6,48], it is extremely important to dissect their relative role and activation mechanisms in both processes.

### Downstream targets of Ca^2+^ in Ca^2+^-induced autophagy

From the above, it is clear that IP_3_-induced Ca^2+^ release can induce autophagy. However, the Ca^2+^-dependent mechanisms involved and the target(s) of the intracellular Ca^2+^ signal remain elusive, although different mechanisms have already been proposed (Figure 1).

Based on experiments using inhibitors of CaMKKβ (STO-609) or of AMPK (compound C) as well as siRNA-mediated knock down of these enzymes, it was proposed that autophagy induced by thapsigargin or other Ca^2+^-mobilizing agents was mediated via CaMKKβ, thereby activating its downstream target AMPK [66,79,85]. The latter is a negative regulator of mTOR while a positive regulator of ULK1, both resulting in the induction of autophagy. However, since thapsigargin also induced mTOR inhibition and autophagy in AMPK knock-out cells [69], although to a lesser extent, an AMPK-independent pathway for autophagy induction is also likely present. In follow up of this study, a recent study elegantly demonstrated that CaMKI was also activated and played a role in autophagy [81]. In particular, it was shown that CaMKI stimulated the formation autophagosomes in a pathway involving PtdIns3K Complex III but independently of AMPK (Figure 1). In this respect, it is important to mention that Vps34, a component of the PtdIns3K Complex III, was already reported to be activated by Ca^2+^ and calmodulin [86], although this finding was later disputed [87].

In a study using hepatocytes and fibroblasts, thapsigargin induced autophagy through ER stress without inhibiting mTOR activity. Phosphorylation of protein kinase C θ (PKCθ) was hereby critical [67]. Treatment with BAPTA-AM decreased both PKCθ phosphorylation and autophagy, demonstrating that Ca^2+^ is needed for PKCθ phosphorylation, though the mechanism remains elusive. Preliminary data indicate that the phospholipase C inhibitor U73122 partially inhibited ER stress-induced autophagy [67], underpinning a role for the IP_3_R. Interestingly, in amino acid starvation-induced autophagy no role for PKCθ was found, suggesting that this pathway is preferentially used during ER stress.

In mesangial cells, Cd^2+^ treatment triggers Ca^2+^ release from the ER, putatively by activation of the IP_3_R, and induces both autophagy and apoptosis. Extracellular signal-regulated kinase (ERK) activation was observed, and inhibition of ERK selectively suppressed the autophagic response, but not the apoptotic cell death response [70]. As ERK activity was inhibited by BAPTA-AM, this study suggests a role for ERK in Ca^2+^-induced autophagy, although the target of ERK was not elucidated. It is hereby interesting to note that ERK may be involved in the phosphorylation of Bcl-2, thereby leading to Beclin 1 release from Bcl-2 [28,88]. Moreover, previous studies had also demonstrated that ERK can be involved in autophagy through regulation of Gα-interacting protein [89] or of the microtubuli [90].

The induction of autophagy by cytosolic Ca^2+^ may therefore depend of one or more of these mechanisms, but the situation might also be more complex, as several other proteins regulating autophagy are known to be influenced by Ca^2+^. These proteins include DAPK, which is regulated by calmodulin and which can phosphorylate Beclin 1, thereby dissociating it from Bcl-2 and inducing autophagy [25]. Finally, members of the S100 Ca^2+^-binding protein family [40] as well as eEF-2 kinase, responsible for the phosphorylation of eukaryotic elongation factor (eEF)-2 [91] have also be implicated in the regulation of autophagy.

Taken together, these data indicate that an induction or a regulation of autophagy by Ca^2+^ is very plausible and that depending on the cell type or the cell state various mechanisms can be involved.

## Conclusion

In conclusion, the available data indicate that ER Ca^2+^- store content, the IP_3_R and IP_3_-induced Ca^2+^ release act on the autophagic process. Under normal conditions, a basal level of IP_3_-induced Ca^2+^ release from the ER to the mitochondria is responsible for a certain level of ATP production, sufficient for keeping AMPK inactive and therefore precluding induction of autophagy. If Ca^2+^ transfer to the mitochondria decreases below a certain threshold, ATP synthesis is not anymore guaranteed, AMP/ATP ratio increases and AMPK is activated, leading to autophagy. Under stress conditions however, Beclin 1 may interact with the IP_3_R, leading to an increased IP_3_-induced Ca^2+^ release. At least part of the released Ca^2+^ diffuses to the cytosol where it can stimulate the induction of autophagy via a not yet completely understood pathway. This pathway may either involve CaMKKβ and AMPK, or develop in an AMPK-independent way. Whatever the mechanism, this dual regulation of autophagy by the IP_3_R and Ca^2+^ is of paramount importance for the determination of cell fate. As autophagy is important in pathological situations as e.g. cancer and neurodegenerative diseases [1,6,8], the correct understanding of autophagy regulation by Ca^2+^ may lead to important therapeutical consequences.

## Abbreviations

2-APB, 2-aminoethoxydiphenyl borate; a.a., Amino acid; AMPK, AMP-activated kinase; ATG, Autophagy genes; BAPTA-AM, 1,2-Bis(2-aminophenoxy)ethane-N,N,N′,N′-tetraacetic acid tetrakis(acetoxymethyl ester); BI-1, Bax inhibitor-1; Bcl-2, B-cell lymphoma 2; CaMKI, Calmodulin-dependent kinase I; CaMKKβ, Calmodulin-dependent kinase kinase β; DAPK, Death-associated protein kinase; ER, Endoplasmic reticulum; ERK, Extracellular signal-regulated kinase; IP3, Inositol 1,4,5-trisphosphate; IP3R, Inositol 1,4,5-trisphosphate receptor; JNK1, c-Jun NH2-terminal kinase-1; LC3, Microtubule-associated protein 1 light chain 3; mTOR, Mammalian target of rapamycin; NAF-1, Nutrient-deprivation autophagy factor-1; PtdIns3K, Phosphatidylinositol 3-kinase; PKC, Protein kinase C; TKO Cells, IP3R triple knock out chicken DT40 B lymphocytes; ULK1, Unc-51-like kinase 1; XeB, Xestospongin B.

## Competing interests

The authors declare that they have no competing interests.

## Authors’ contributions

JBP, JPD and GB wrote the manuscript. All authors approved the final version.
